# Phone Calls Over Clicks: A Survey Study Assessing Nurses’ Communication Preferences During Inpatient Physician Rounds

**DOI:** 10.1007/s11606-025-09805-y

**Published:** 2025-10-01

**Authors:** Prabhava Bagla, Jasmah Hanna, John Hanfelt, Stacey Watkins

**Affiliations:** 1 Emory University School of Medicine, Department of Medicine, Division of Hospital Medicine, 80 Jesse Hill Jr Dr SE, Atlanta, GA 30303 USA; 2 Emory University Department of Biostatistics and Bioinformatics, 1518 Clifton Road, Atlanta, GA 30322 USA; 3 Grady Memorial Hospital, Hospital Medicine, Atlanta, USA

## Background

Communication among physicians and nurses is a crucial part of safe and effective healthcare delivery. According to the Joint Commission, miscommunication continues to be a leading contributor to sentinel events, consistent with findings from previous years^1^. At our large, tertiary care academic hospital, various modes of communication exist in the inpatient setting: in-person discussions, pages from nurses, phone calls from physicians to nurses (as such or in response to pages), and chat functionality in our electronic medical record (EMR) by Epic. Healthcare providers utilize a mix of these throughout the workday. The Situation, Background, Assessment, and Recommendation format is already widely used at our institution to maintain standardized content of communication^2^. In response to a request from nursing leadership for improved communication, we sought to objectively assess nurses’ perception of the current state of physician-nurse communication at our hospital.

## Methods

We selected three units comprising 30 individual patient beds each. These units operate under unified leadership at the nurse manager level, facilitating project buy-in. We sought responses from the 53 dayshift nurses working on these units. Emory University Institutional Review Board considered this to not be human subjects research.

We created an anonymous online survey; it included demographic questions followed by items focused on nursing perception of communication around the time of provider rounding, rated on a 5-point Likert scale from “poor” to “excellent”; how often physicians contacted them before or after rounding via phone call, and how often they had to reach physicians after rounds to inquire about the plan of care. Surveys were emailed to nurses via their unit directors. Nurses were subsequently reminded via email and in-person interaction over the next 2 months to encourage responses.

Statistical analysis was conducted via chi-square exact tests. *P*-values less than 0.05 were regarded as significant. Statistical analysis was supported by NIH/NCATS grant UL1TR002378.

## Results

Out of the 53 nurses surveyed, 40 responded, yielding a 75.47% response rate. Table 1 shows the results of the survey. Statistical analysis revealed that the frequency with which physicians communicate with nurses after seeing the patient was significantly associated with higher communication ratings (*p* = 0.005). Additionally, the frequency of physician communication with nurses before seeing patients approached statistical significance (*p* = 0.07).

**Table 1:** Nurses’ Characteristics and Their Association with Perception of Physician-Nurse Communication

	Physician-nurse communication			
	Total	Poor	Fair or better	*P-*value
Gender				*P* = 0.15
Female	29 (73%)	15 (52%)	14 (48%)	
Male	6 (15%)	4 (67%)	2 (33%)	
Prefer not to say	5 (13%)	5 (100%)	0 (0%)	
Age (years)				*P* = 0.38
25– 30	18 (45%)	13 (72%)	5 (28%)	
31 – 40	13 (33%)	7 (54%)	6 (46%)	
41 and above	9 (23%)	4 (44%)	5 (56%)	
Years of nursing				*P* = 0.52
0 – 3	6 (15%)	4 (67%)	2 (33%)	
4 – 6	18 (45%)	11 (61%)	7 (39%)	
7 – 10	8 (20%)	6 (75%)	2 (25%)	
11 and above	8 (20%)	3 (38%)	5 (63%)	
Race				*P* = 0.66
Black or African-American	25 (63%)	14 (56%)	11 (44%)	
Other race	7 (18%)	4 (57%)	3 (43%)	
Prefer not to say	8 (20%)	6 (75%)	2 (25%)	
How often do physicians speak with you before rounding on a patient?				*P* = 0.07
Never	14 (35%)	10 (71%)	4 (29%)	
Rarely	20 (50%)	13 (65%)	7 (35%)	
Sometimes or often	6 (15%)	1 (17%)	5 (83%)	
How often do physicians speak with you after rounding on a patient?				*P* = 0.005
Never	17 (43%)	14 (82%)	3 (18%)	
Rarely	13 (33%)	8 (62%)	5 (38%)	
Sometimes or often	10 (25%)	2 (20%)	8 (80%)	

## Discussion

Our study demonstrated that despite the availability of multiple communication channels, including EMR-based chat, phone calls remain preferred for nurse-physician interaction during rounds. Infrequency of such calls may contribute to the perceived poor overall quality of communication at our facility.

Several researchers have investigated approaches to improve physician–nurse collaboration, from notification systems alerting nurses about upcoming rounds^3^ to standardized scripts for bedside multidisciplinary rounds^3^, ^4^. A major limitation of such approaches is the time constraint preventing nurses’ participation in bedside rounds while managing multiple patients simultaneously. Our study focused on determining how physicians can most effectively communicate with nurses during rounds to establish a shared understanding of recent events and future plans.

Communication methods in our hospital can be classified based on real-time interaction capability, each with advantages and disadvantages (Table 2). While asynchronous methods like EMR messaging offer convenience and documentation, our results suggest real-time synchronous communication maintains substantial value. This preference likely stems from phone calls enabling immediate clarification, facilitating complex discussions, and providing opportunities to share observations not captured in the EMR. Voice communication may also strengthen interprofessional relationships beyond text-based methods.

**Table 2:** Modes of Communication Between Physicians and Nurses

Mode of communication	Real-time interaction	Pros	Cons
In-person	Yes	Easy Not dependent on technology	Time and availability constraints
Phone call	Yes	Easy Limited dependence on technology	Availability constraints Phone number availability
Page	No	Reply at convenience	No two-way communication Delay in response Availability of recipient to answer
EMR chat	Yes	Easy Reply at convenience	Dependent on technology Delay in response

Our study had some limitations. Firstly, we were unable to determine if nurses’ perception of quality of communication correlated with patient outcomes. Secondly, as this was a single-center study, our results may not be generalizable to other settings. However, our high survey response rate indicates robust internal validity and strong nursing engagement in addressing communication challenges.

The findings of this study highlight continued preference for real-time synchronous communication even in an increasingly digital healthcare environment. Our future research will focus on developing and evaluating interventions to facilitate physician-nurse phone communication to enhance collaboration, ultimately improving patient care and safety.
